# Low-dose hydroxycarbamide therapy may offer similar benefit as maximum tolerated dose for children and young adults with sickle cell disease in low-middle-income settings

**DOI:** 10.12688/f1000research.14589.1

**Published:** 2018-09-04

**Authors:** Baba Psalm Duniya Inusa, Wale Atoyebi, Abdul Aziz Hassan, Tushar Idhate, Livingstone Dogara, Ifeoma Ijei, Yewen Qin, Kofi Anie, Juliana Olufunke Lawson, Lewis Hsu

**Affiliations:** 1 Paediatric Haematology, Evelina London Children’s Hospital, Guy’s and St Thomas NHS Foundation Trust, London, UK; 2 Department of Haematology, Oxford University Teaching Hospital, Oxford, UK; 3 Department of Haematology & Blood Transfusion, Faculty of Basic Clinical Sciences, College of Health Sciences, Ahmadu Bello University & ABU Teaching Hospital, Zaria, Nigeria; 4 Division of Paediatric Haematology and Oncology, Department of Paediatrics, Mahatma Gandhi Mission Medical College and Hospital, Aurangabad, India; 5 Haematology and Blood Transfusion, Faculty of Clinical Sciences, Kaduna State University College of Medicine, Kaduna State University, Kaduna, Nigeria; 6 Paediatrics Department, University Hospital, Lewisham and Greenwich NHS Trust, King's College London, London, UK; 7 Haematology and Sickle Cell Centre, London North West University Healthcare NHS Trust, London, UK; 8 Imperial College London, London, UK; 9 Department of Paediatrics, Zankli Medical Centre, Abuja, Nigeria; 10 Pediatric Sickle Cell Center, University of Illinois at Chicago, Chicago, Illinois, USA

**Keywords:** sickle cell disease, hydroxyurea, hydroxycarbamide, low-middle income countries, anaemia

## Abstract

The multiple clinical benefits of hydroxycarbamide in sickle cell disease are supported by a large body of evidence. The maximum tolerated dose (MTD) is the regimen recommended by guidelines from a panel of National Heart, Lung, and Blood Institute (NHLBI) experts, but other dosage regimens have been used in babies (BABY-HUG) 9 to 18 months old (20 mg/kg per day) and developing countries such as India (10 mg/kg per day); however, there has been no direct comparison of the efficacy, effectiveness, or cost-effectiveness of these different regimens. The purpose of this review was to investigate the current situation with various hydroxycarbamide regimens with particular relevance to low-middle-income countries. In regard to methodology, a literature review was undertaken by using multiple databases in PubMed and Google and the search terms included sickle cell disease, hydroxyurea, hydroxycarbamide, sickle cell anaemia, low-middle-income countries, Sub-Saharan Africa, and India. Although MTD regimens have been widely used in research, especially within North America, clinical trials elsewhere tend to use fixed-dose regimens. In a survey of haematologists across Europe and Africa, 60% (75% response rate) did not use the MTD regimen for hydroxycarbamide treatment of sickle cell disease. The recommendations are (1) for practical purposes to commence using fixed-dose hydroxycarbamide in line with BABY-HUG recommendations and then (2) to consider or propose a trial comparing MTD escalation with various fixed doses and to include as end points health-related quality of life, haemoglobin F levels, adherence, and cost-effectiveness.

## Introduction

Sickle cell disease (SCD) is an inherited autosomal recessive genetic disorder caused by the presence of haemoglobin S (HbS) ^1, 2^. SCD is one of the most common genetic diseases in the world ^3^. It is estimated that there are over 300,000 annual births with SCD globally and that 80% are from Sub-Saharan Africa ^4^. In the UK, 1 in about 2,000 babies (300 babies annually) is born with SCD compared with over 100,000 annual births with SCD in Nigeria ^5– 7^. This condition is associated with lifelong risk of frequent hospitalisation, poor quality of life, and decreased life expectancy ^1^. Large gaps exist for SCD survival and care between high-income countries and low-middle-income countries (LMICs) (over 99% in East London will survive into adulthood, but less than 50% in Sub-Saharan Africa will reach their 10th birthday) ^8, 9^. Treatment for SCD, despite perhaps being the most prevalent inherited life-threatening disorder, has not received high priority in many countries ^6, 10^.

## Clinical complications of sickle cell disease

The homozygous state (HbSS) is referred to as sickle cell anaemia (SCA) and accounts for over 70% of SCD in the UK ^11^. Other forms of SCD include co-inheritance of HbS with haemoglobin C (HbSC), which accounts for about 20% of SCD and generally is less severe than SCA. The central pathologic feature occurs in a deoxygenated state when haemoglobin undergoes paracrystal formation and affected red blood cells (RBCs) become distorted, developing into the characteristic “sickle” shape ^2^. These RBCs interact with other vascular entities, leading to further obstruction of small vessels, tissue ischaemia and infarction, and haemolysis. A complex cascade of vascular and endothelial reactions occurs, leading to further tissue damage ^12^. In early childhood, SCD presents with clinical complications, including acute pain, anaemia, and end organ damage (for example, 11% with overt stroke by 18 years ^13^ and silent cerebral infarction in over 30% by age 15 ^14^).

The hallmark of SCD is the occurrence of painful vaso-occlusion episodes, sometimes known as a “pain crisis” ^15^. This occurs when deformed erythrocytes (due to paracrystal formation) occlude the microvasculature, resulting in tissue infarction, oedema, and localised inflammation subsequent to downstream tissue anoxia ^16, 17^. This can lead to necrosis of the end organs and tissues, and necrosis of the bone marrow is most frequent. Often multiple sites of bone are affected, so patients may present with “dactylitis” pain and/or swelling of the hands and feet ^9^ or sudden generalised pain ^18^. Another complication is acute chest syndrome (ACS), which has a high mortality rate in children and adolescent patients with SCD ^18^. ACS is defined as (1) the presence of a new infiltrate on chest X-ray associated with (2) one or more symptoms of fever, cough, excess sputum, dyspnoea, or hypoxia ^19^. In order to reduce the severity of the disease, the most common preventive intervention before medications for SCD became available was regular blood transfusions ^13^.

### Hydroxycarbamide utilisation in sickle cell disease

The drug known as hydroxycarbamide (HC) in the UK and hydroxyurea (HU) in the rest of the world is a ribonucleotide reductase inhibitor ^20^. The role of HC therapy in SCD was first conclusively proven in adults in the landmark “Multicenter Study of HU in Patients with Sickle Cell Anaemia”, a randomised double-blinded placebo-controlled trial using a maximum tolerated dose (MTD) regimen ^21^. The rate of sickle cell crises was 44% lower in the HC group than in the placebo group. Other complications that showed significant reduction were ACS cases and the need for blood transfusion. Additional data suggest an overall 40% reduction in mortality in the HC group, but the evidence is not rigorous ^22^.

In the phase I/II HUG-KIDS trial ^23, 24^ and then the BABY-HUG phase III clinical trial ^21, 25^, HC benefits in infants and children were reaffirmed to be similar to those observed in adult SCD. Additional safety factors of the drug were also investigated, and it was shown that HC did not seem to present any serious inhibition of child growth and development ^23^. The phase III BABY-HUG trial was a randomised double-blinded placebo-controlled trial with a larger sample population of 193 patients (9 to 18 months old) with either HbSS or HbSβ ^0^ thalassaemia but did not examine disease severity ^25^. Results of the BABY-HUG study supported earlier work; HC could be implemented even earlier in life, and there were no serious adverse effects ^25, 26^. Acute events were significantly reduced by HC, but protection from organ damage to the spleen and kidney was not shown in the BABY-HUG study. The other important conclusion from BABY-HUG was that administering HC could be beneficial for patients who had not presented with severe manifestations of SCD in a prophylactic manner, rather than only for patients with multiple previous cases of pain crises or other complications. A “brain protection” indication for HC was shown in the landmark TWITCH (TCD With Transfusions Changing to Hydroxyurea) study, showing that HC therapy can be comparable to chronic blood transfusion in selected children at high risk for stroke ^27^.

Improving health for young people with SCD has the potential to promote an individual’s well-being, reduce hospital admissions, and improve educational achievement ^25, 28^. A number of systematic reviews and US National Institutes of Health (NIH) guidelines recommend increased utilisation of HC in SCD ^12, 29, 30^. HC was approved by the US Food and Drug Administration for adults in 1998 ( https://www.fda.gov/Drugs/InformationOnDrugs/ApprovedDrugs/ucm590096.htm). NIH guidelines in the US recommend that providers “offer hydroxyurea for children with SCA starting at 9 months of age” ^31^. The British Society Of Haematology have recently published UK guidelines for the use of Hydroxycarbamide in adults and children, these have been summarised ( Supplementary File 1) ^32^.

HC has multiple mechanisms of benefit for SCD. It increases foetal haemoglobin (HbF) expression, and the HbF blocks HbS polymerisation. HC reduces the quantities of platelets and white blood cells, further improving blood flow in patients with SCD ^33^. HC improves RBC hydration and flexibility and decreases abnormal adhesion between blood cells and the blood vessel wall. HC increases the formation of nitric oxide, which in turn stimulates guanylate cyclase activity ^34, 35^, which also enhances blood flow. HC is also reported to improve spleen and renal function ^36^. Together, these combine to produce the clinical and laboratory improvement observed in SCD.

The toxicities in HC therapy may be grouped according to short-term, medium-term, or long-term concerns ^22^. Short-term complications are predominantly haematological toxicities such as cytopenias. HC is an alkylating cytotoxic agent which is associated with dose-dependent marrow suppression. The main mechanism of action of HC is the suppression of mature erythrocytes and the increase in younger erythrocytes, which have a greater HbF production ^36^. Cytopenia due to HC is not selective to erythrocytes and may therefore lead to the risk of pancytopenia involving leucopoenia, increased risk of immunosuppression, thrombocytopenia, and reticulocytopenia.

Medium-term toxicity is reported with decreased sperm cells (azoospermia and oligospermia ^23, 37^), which might be transient. It should be noted that baseline sperm abnormalities are frequent in males with SCD, and rates are as high as 91% ^37– 40^. It could potentially alarm carers/parents and patients, hence the reluctance to accept the use of HC. An unproven lingering concern is the risk of secondary neoplasms ^29^, but recent studies on HC in myelodysplastic states show no increased risk of cancer ^21^.

## Hydroxycarbamide regimens in practice

In addition, the authors conducted a survey of HC practice patterns in haematologists and paediatricians across the European Union and Sub-Saharan Africa in 2017. The response rate was 75%, representing 53 sites. The results ( Table 1 and Table 2) reveal that only 28% use the MTD as against 30% who base their treatment dosage on minimum effective dose whereas almost 45% are happy to randomly assign HC versus placebo in a clinical trial setting. Clinicians’ attitudes, though, might have changed since the time of that survey in 2017.

**Table 1.** Starting hydroxycarbamide therapy in sickle cell disease.

What is the youngest age at which you would start treatment?		
Answer	Count	Percentage
0–6 months (A1)	2	3.85%
6 months–2 years (A2)	22	42.31%
2–5 years (A3)	10	19.23%
5–10 years (A4)	1	1.92%
More than 10 years (A5)	2	3.85%
Other	5	9.62%
No answer	1	1.92%
Not completed or not displayed	9	17.31%

**Table 2.** Designing a clinical trial in sickle cell disease.

Would you be willing to randomly assign young children to hydroxycarbamide or placebo in a trial?		
Answer	Count	Percentage
No (A1)	4	7.69%
Yes, children	6	11.54%
Yes, children 6 months–2 years	23	44.23%
Yes, children 2–5 years (A4)	23	44.23%
Yes, children 5–10 years	21	40.38%
Yes, children older than 10 years	21	40.38%
Other	6	11.54%
Not completed or not displayed	10	19.23%

**Table:** 

In a trial to study the effect of hydroxycarbamide in protecting against ischaemic cerebral damage, what dosage scheme would you prefer to use?		
Answer	Count	Percentage
Standard fixed dose (A1)	11	21.15%
Escalation to maximum tolerated dose (A2)	18	34.62%
No preference (A3)	12	23.08%
No answer	1	1.92%
Not completed or not displayed	10	19.23%

## Study goal

The overarching question is whether low-dose HC will offer a benefit similar to that of MTD HC in children and young adults with SCD, especially in low-middle-income settings.

i) Is adherence to HC therapy significantly greater with a fixed low dose (FLD) than an MTD HC regimen?

ii) Is health-related quality of life (HRQOL) significantly greater with an FLD than an MTD HC regimen?

iii) Are the total health and social care costs of caring for this population significantly lower with an FLD than an MTD HC regimen and is the FLD regimen more cost effective?

iv) It is also possible that early onset initiation of HC treatment leads to more sustained retention of HbF and F cells ^41^.

In several studies, the choice of FLD HC seems to arise partially from theoretical concerns that HC therapy would cause significant immunosuppression and allow tropical diseases to overwhelm the patient with SCD, and so a lower dose was considered safer than MTD HC for initial clinical trials. In India, Uganda, and Nigeria, rates of malaria did not increase in the patients on HC at fixed dose ^21, 42– 45^. In the Nigerian study, 1 patient out of 28 had pulmonary tuberculosis reactivation after HC therapy, but it is not clear whether this is a greater risk than the background rate of tuberculosis ^46^. As of this writing, studies have not examined the risks of tropical infectious diseases with HC at MTD.

## Literature search methodology

We used a combination of databases, including Medline (using PubMed query tools), Cochrane review, and Google search engines, to identify the following search terms: hydroxyurea/hydroxycarbamide, sickle cell disease, sickle cell anaemia, low-middle-income setting, Sub-Saharan Africa, and India.

## Results

Clinical trials have reported benefits for MTD HC and FLD HC, as summarised in a recent Cochrane review ^15^. The majority of existing clinical trials of HC have relied on the use of the MTD regimen where an incremental dosing system is adopted until a dose is achieved just below the onset of toxicity. The rationale for a MTD regimen is that pharmacokinetics and marrow tolerance for HC have high variation between individuals, leading to a “personalised medicine” approach ^15^. The advantage of the MTD strategy is providing the patient with the maximal clinical benefit of HC administration with a personalised dose, and this MTD HC has been integrated into the management regimen for treating SCD, particularly in high-income countries ^36^. The disadvantages of MTD are that patients may experience unwarranted side effects plus the burden of frequent blood tests and HC dose adjustments. This burden on the patients might account for low acceptance of HC. Other patients might not feel clinical benefit during the dose-escalation period and hence might give up on HC before attaining MTD.

A regimen of FLD HC was exemplified by the BABY-HUG study, which randomly assigned children (9 to 18 months old) to a fixed dose of 20 mg/kg HC or placebo ^19^. The advantages of a low dose are that patients may experience less toxicity such as reduced risk of myelotoxicity and other dose-related toxicity and hence may not require frequent monitoring compared to that required for the MTD regimen. This implies that the FLD HC regimen will cost less than the MTD regimen for the patients and the health-care system (fewer lab tests and clinic visits). The disadvantage of low-dose HC is that some patients might not achieve the full clinical benefits of treatment.

## Adherence

Taking HC consistently is necessary for effective treatment. Lack of adherence is widely recognised as a challenge ^47^. One factor that contributes to poor adherence is absence of immediate “feeling of benefit” when an HC dose is taken or subjective worsening if a dose is missed. In addition, HC only decreases severity of SCD but is not a complete cure, and so even a patient taking HC consistently can be disappointed to find that they still suffer complications of SCD. HC capsules have relatively easy storage requirements, but HC does need protection from excessive heat and direct sunlight (per HC package insert). Young children might need HC as a palatable liquid or as dissolving tablets, and access to these formulations might be more limited than to capsule formulations.

Techniques have been developed to encourage good adherence to HC therapy, which could be applied to an LMIC context. Patients can be given encouragement verbally at frequent clinic visits. They can receive visual feedback that the HC effect is seen in their blood test results or long-term clinical course over several months ^15^. Medication bottle caps have been created with sensors to detect whether the bottle is opened daily. Cell phone apps have also been developed to provide encouragement (automated or human) and directly observed therapy by “selfie” photo ^48^. Additional personal monitoring techniques include assigning a peer mentor, patient navigator, or community health worker ^49^. No information could be found comparing adherence to MTD and FLD HC regimens.

Health Related Quality of Life (HRQOL) as measured in multiple dimensions improves with HC treatment whether with a MTD or a fixed low dose ^50, 51^. Moreover, HRQOL is generally accepted to incorporate different domains such as an individual’s physical health, psychological state, social relationships, levels of independence, and relationship to important aspects of their environment. HRQOL focuses on how an individual’s illness and treatment impacts different domains of their life and is not restricted to their physical functioning ^52^. For example, the PedsQL™ 4.0 is a multi-dimensional measure covering physical functioning, emotional functioning, social functioning, and school functioning, which includes three summary scores (total, physical health, and psychosocial health). The PedsQL™ can be used as a child self-report measure for children 5 to 18 years old and as a parent proxy measure for children 2 to 18 years old. Evidence suggests that the PedsQL™ is valid, reliable, and sensitive to changes in a child’s condition over time ^53^ and sensitive to cognitive development. In addition, the PedsQL™ has been found to differentiate between paediatric SCD populations and healthy populations ^54, 55^. SCD can significantly harm HRQOL both acutely and chronically. HRQOL measures that were developed in the US or the UK might need to be validated for rigorous application in other countries and other languages.

## Costs

Economic benefits have been reported with both regimens ^25, 42, 56, 57^. It is possible that an FLD with improved adherence in fact produces more economic benefits because the costs of surveillance are lower. Many of the studies conducted in Africa and India used the FLD strategy ^51– 54^. HC is not inherently expensive (for example, USD $1 for an adult on an FLD regimen in Nigeria) ^46^. However, the cost of daily HC might be a barrier for people with low incomes and no health insurance coverage.

## Global implementation of hydroxycarbamide to treat sickle cell disease

HC could be expected to play a major role in global SCD treatment because, of all the currently available management regimens, this may be the most straightforward to successfully initiate in developing countries. The basis on which this claim is made is that it is the only therapy option that, in light of current research, has been shown to be actually capable of reducing the severity of SCD complications via a single intervention. Compared with other management options like blood transfusion, HC is the simplest to implement in a population; it is a once-daily oral therapy ^24^. This is a pragmatic plan that is particularly relevant to developing countries because of the often-low investment in resource availability.

HC is probably more widely available across the world than other therapeutic options for SCD. The majority of developing countries do not have a robust blood-banking infrastructure for chronic blood transfusion and have limited access to haematopoietic stem cell transplant and pharmaceutical-grade L-glutamine ^45^.

Stroke and other cerebrovascular damage are common in childhood SCD and are among the most devastating complications of SCD. The “brain protection” indication for HC ^47, 48^ would be important in many LMICs where chronic blood transfusions face greater problems with safety and availability than in the blood banks of high-income countries ^58^. Another HC indication that has received little attention has been priapism, but the potential to decrease suffering and impotence could be significant for quality of life ^26^.

The more economically developed countries such as the US and the UK have designated SCD as a public health issue and so the quality of care is rising, which in these countries has meant that mortality rates in young patients have been lowered to less than 5% ^31^. Sub-Saharan Africa, which carries the highest burden of SCD morbidity and mortality, also lags in medical resources and expertise. Although new advances such as HC are welcomed (and understandably so), the inevitable question is whether the “larger picture” is being missed in Sub-Saharan Africa. In light of this discrepancy in the availability of medical knowledge and resources, there have been calls to increase and improve the sharing of research between developed and developing countries. It is hoped that, when such networking is stimulated, a more rounded understanding of the condition and its worldwide effects could be established, such as further investigation into the genetic diversity and environmental effects of SCD. Capacity-building around SCD can potentially stimulate broader and deeper biomedical resources in LMICs. In view of the issues and concerns that have been clearly documented in this paper in relation to HC access, costs and supportive care, we have proposed recommendations for the initiation, dosing and monitoring of HC in LMICs ( Supplementary File 2). This will provide a template for further discussion and ultimately collaborative efforts involving clinical trials and international guidelines.

## Conclusions

The use of HC has shown favourable results in terms of improving patient outcomes and safety in adults and children, and so this intervention method provides a promising facet in the improvement of patient survival in SCD. However, one must remember the worldwide scale of the condition and how, in countries and locations where the disease is prevalent, management is often inadequate. Thus, the avenues of widening scientific collaboration between countries and their research may be an even more pressing issue, one that could form the framework for future developments in this ever-progressing field.

## Supplementary material

**Supplementary File 1. Summary of recommendations and an algorithm for the use of HC in adults and children with SCD from a British Society for Haematology guideline.** Based on review of current evidence, this guideline provides graded recommendations on indications for initiating HC as well as monitoring of effectiveness and toxicity. It aims to provide a platform for joint clinician and patient decision making as well as improve equity of access.

**Supplementary File 2. Recommendations for initiation, dosing and monitoring of HC in low middle income settings.** In recognition of the well documented limited access, cost and supportive care in low middle class countries, we provide suggested recommendations for initiating, the dosage and monitoring of HC mostly applicable to children. It incorporates the use of both the Lowest Effective Dose (LED) and Maximum Tolerated Dose (MTD) dependent on the indication for use.
