# Supplementary material for: Low-dose hydroxycarbamide therapy may offer similar benefit as maximum tolerated dose for children and young adults with sickle cell disease in low-middle-income settings
Source: F1000Res. 2018 Sep 4;7:F1000 Faculty Rev-1407. [Version 1] doi: 10.12688/f1000research.14589.1 (PMC6124375; doi:10.12688/f1000research.14589.1)
Supplement: Supplementary file 1 [file f1000research-7-15876-s0000.tgz › ae390555-de05-4439-9eef-f36818fdea6a_Supplementary_File_1.docx]

**Appendix I**

**Title**

Summary of recommendations and an algorithm for the use of HC in adults and children with SCD from a British Society for Haematology guideline.

**Legend**

Based on review of current evidence, this guideline provides graded recommendations on indications for initiating HC as well as monitoring of effectiveness and toxicity. It aims to provide a platform for joint clinician and patient decision making as well as improve equity of access.

**Recommendations**

- In infants 9 to 42 months old with sickle cell anaemia (SCA), offer treatment regardless of clinical severity to reduce sickle cell complications (pain, dactylitis, acute chest syndrome, and anaemia) (grade 1A)
- In children older than 42 months, adolescents, and adults with SCA, consider treatment regardless of clinical severity to reduce sickle cell complications (pain, dactylitis, acute chest syndrome, and anaemia) (grade 2C)
- The benefits of hydroxycarbamide should be discussed with all parents of children, adolescents, and adults with SCA to enable informed joint decision making, and there should be ongoing discussion between provider and patient (grade 1D)
- Children who have started regular blood transfusions for abnormal transcranial Doppler (TCD) should be considered for hydroxycarbamide therapy (with or without venesection) if they have received at least 1 year of regular transfusions and have no magnetic resonance angiography (MRA)-defined severe vasculopathy (grade 1A)
- In children who are treated for primary stroke prevention who are changing from regular blood transfusions to hydroxycarbamide therapy, transfusion should be continued until they have reached maximum tolerated dose of hydroxycarbamide and TCD velocities should be monitored every 3 months (grade 1C)
- Children with TCD velocities in the range of 170 to 200 cm/s (conditional risk category) should be treated with hydroxycarbamide therapy to help prevent progression from conditional to abnormal TCD velocity (grade 1B)
- When treating children with conditional TCD velocities with hydroxycarbamide, the dose should be escalated to maximum tolerated dose and TCD velocities should be monitored every 3 months (grade 1C)
- In children and adults with a history of acute ischaemic stroke or silent infarcts, hydroxycarbamide should be recommended as second-line therapy for secondary stroke prevention when transfusions are contraindicated or unavailable (grade 1B)
- There are insufficient data to advise commencing hydroxycarbamide therapy for primary stroke prevention in adults (grade 1D)
- In patients with sickle cell nephropathy with persisting proteinuria despite angiotensin-converting enzyme inhibitor/angiotensin receptor blocker therapy, consider the addition of hydroxycarbamide therapy (grade 2C)
- Disease-modifying treatments (hydroxycarbamide or transfusion) should be considered in patients with SCA and pulmonary hypertension and in children with chronic hypoxia (grade 2C)
- In adults (and children) with SCA and symptomatic chronic anaemia that interferes with daily activities or quality of life, treat with hydroxycarbamide (grade 1C)
- There is insufficient evidence to recommend hydroxycarbamide for the prevention or treatment of other chronic disease complications, but its potential risks and benefits should be discussed with the patient (grade 1D)
- Hydroxycarbamide therapy should be considered in adults and children with sickle cell disease (SCD) with genotypes other than SS and Sβ^0^ thalassaemia who have recurrent acute pain, acute chest syndrome, or episodes of hospitalisation (grade 2C)
- Post-pubertal male patients should be offered sperm analysis and cryopreservation prior to starting treatment with hydroxycarbamide (grade 1C)
- Hydroxycarbamide should be discontinued for at least 3 months pre-conception in male and female patients (grade 1C)
- Contraception is advised for patients on hydroxycarbamide (grade 1C)
- Guidelines for the indications, initiation, and monitoring of hydroxycarbamide therapy should be available at all sites caring for patients with SCD (grade 1D)

**Algorithm for hydroxycarbamide dosing and monitoring (UK British Committee for Standards in Haematology Guidelines)**

Neutrophils >1 x 10^9^/l

and/or platelets >80 x 10^9^/l

Monitor FBC monthly

Neutrophils > 1 x 10^9^/l

and/or platelets >80 x 10^9^/l

sustained for 8 weeks

- Once a stable dose is established, laboratory safety monitoring should include FBC and reticulocyte count every 2-3 months. Follow above for dose modifications. HbF and MCV can be used to monitor effect/compliance.
- MCV and HbF levels for evidence of consistent or progressive laboratory response

:

– FBC with WBC differential, reticulocyte count, and platelet count every 2–3 months

Increase hydroxyurea by 5 mg/kg per day until maximum 35 mg/kg per day.

Monitor FBC in 2 weeks after dose increase.

Follow above for dose modifications.

Start hydroxyurea

Adults 15 mg/kg per day (5-10 mg/kg per day if renal impairment eGFR <60 ml/min per 1.73 m^2^) (round to nearest 500 mg)

Children 20 mg/kg per day

Monitor FBC in 2 weeks’ time

Monitor FBC monthly and follow above for dose modifications.

Neutrophils <1 x 10^9^/l

and/or platelets <80 x 10^9^/l

and/or Hb <45 g/l and

retics <80 x 10^9^/l or 20% decrease in Hb

**Stop hydroxyurea and repeat FBC weekly until**

Neutrophils >1.x 10^9^/l in adults

and/or platelets > 80 x 10^9^/l

Hb >45 g/l or >20% decrease

Start same dose if transient drop or reduce by 5 mg/kg per day

eGFR, estimated glomerular filtration rate; FBC, full blood count; Hb, haemoglobin; HbF, haemoglobin F; MCV, mean corpuscular volume.
