# Supplementary material for: Low-dose hydroxycarbamide therapy may offer similar benefit as maximum tolerated dose for children and young adults with sickle cell disease in low-middle-income settings
Source: F1000Res. 2018 Sep 4;7:F1000 Faculty Rev-1407. [Version 1] doi: 10.12688/f1000research.14589.1 (PMC6124375; doi:10.12688/f1000research.14589.1)
Supplement: Supplementary file 2 [file f1000research-7-15876-s0001.tgz › 593e672a-7fa4-45ff-abf5-a19cfb56068d_Supplementary_File_2.docx]

**Appendix II**

**Title**

Recommendations for initiation, dosing and monitoring of HC in low middle income settings.

**Legend**

In recognition of the well documented limited access, cost and supportive care in low middle class countries, we provide suggested recommendations for initiating, the dosage and monitoring of HC mostly applicable to children. It incorporates the use of both the Lowest Effective Dose (LED) and Maximum Tolerated Dose (MTD) dependent on the indication for use.

**A recommended dose**

The therapeutic dose range of hydroxycarbamide is 15–35 mg/kg daily, and for some indications clinical response at the lower end of the range is sufficient (lowest effective dose), whilst for other indications, particularly involving cerebrovascular disease, the dose is escalated to the higher end of the range or until myelosuppression occurs (maximum tolerated dose, or MTD).

Statement in correspondence about MTD aim to highlight need for close attention to results?

Most children start at a dose of 20 mg/kg daily (to the nearest 100 mg) unless there is particular concern about the risk of myelosuppression, when lower doses should be used.

**Dose adjustment and monitoring**

For most patients, the dose is increased by 5 mg/kg every 2–3 months until there is evidence of clinical benefit, which is the lowest effective dose.

Full blood count (FBC), reticulocytes, renal and hepatic function, and foetal haemoglobin percentage should be checked 2 weeks after starting, and after any dose increase, until the dose is stable and then every 8 weeks to 3 months.

Assess clinical response and if suboptimal increase by 5 mg/kg every 8 weeks until target ranges are reached:

- Neutrophils 2.0–4.0 × 10^9^/l
- Platelets ≥100 × 10^9^/l
- Reticulocytes ≥1.5% or 100 × 10^9^/l

If cytopenias occur, a dose adjustment should be made; see table below for dose adjustment for haematological toxicity. This is particularly important in patients who are more likely to experience myelosuppression, where the aim is to increase the dose to MTD.

| Neutrophils  (×10^9^/l) | Reticulocytes | Platelets  (×10^9^/l) | Dose adjustment |
| --- | --- | --- | --- |
| ≥1.5 | ≥1% or  ≥100 × 10^9^/l | ≥80 | Continue current dose |
| <1.5 | <1% or  <100 × 10^9^/l  <50 × 10^9^/l | <80 | Stop treatment and recheck FBC twice weekly until neutrophils >1.5 × 10^9^/l and platelets >80 × 10^9^/l  Restart at lower dose: reduce by   - 2.5 mg/kg per day or - 500 mg/day (1 capsule) or - 100 mg/day (Siklos 1 capsule)   Stop hydroxycarbamide for 2 weeks or until FBC has recovered and then restart at the dose prior to the recent escalation. This is the MTD. |

Other toxicities:

Renal: increase in serum creatinine ≥50% baseline

Hepatic: >100% increase in alanine aminotransferase

Stop hydroxycarbamide, contact the family directly with instructions, and arrange further tests to monitor recovery.

**Dose adjustment to maximum tolerated dose for patients with cerebrovascular disease**

For some indications, such as those involving cerebrovascular disease, the dose should be increased every 6–8 weeks by increments of 5 mg/kg per day to a maximum of 25–30 mg/kg per day (maximum dose 2,000 mg) or until limited by myelosuppression (MTD):

Target blood results for MTD:

Target neutrophil count 2.0–4.0 × 10^9^/l

Total daily dose 25–30 mg/kg per day

If neutrophils <1.0 × 10^9^/l, platelets <80 × 10^9^/l, or reticulocytes <50 × 10^9^/l, discontinue for 2 weeks or until recovered and restart a lower dose (usually the dose prior to the most recent dose increase).

Parents should also be advised to bring the child to hospital for assessment and urgent blood tests if they develop symptoms suggestive of sepsis, or unusual bruising or bleeding, because of the possible risk of bone marrow suppression.

Inform the general practitioner/shared care hospital team in writing of any dose adjustments and blood test results.

**Admission to hospital**

Hydroxycarbamide should be continued during admission unless the blood results indicate bone marrow suppression or the patient is septic.

**Withdrawal of hydroxycarbamide**

Patients should usually be treated for at least 6 months before deciding to stop hydroxycarbamide because of lack of benefit. When hydroxycarbamide is associated with clinical improvement, it is typically continued for at least 2–3 years; consideration is then given to stopping it, depending on the initial indications, the views of the child and caregivers, and circumstances at that time. In general, it is better to stop hydroxycarbamide during school holidays and not just before important events such as exams.
